# Remarks on Dr. M'Kinnal's Report of the Yellow Fever on Board the Sybille Frigate
*The whole of Dr. M'Kinnal's Report has not been published. An analysis of it was given in the last Number of the Med.-Chir. Review.—Editor.


**Published:** 1831-01

**Authors:** 


### YELLOW FEVER.

### *Remarks on* Dr. M'Kinnal's *Report of the Yellow Fever on Board the Sybille Frigate.** By Sanitarius.

*To the Editor of the London Medical and Physical Journal.*

Sir: The readiness and liberality with which the naval medical commissioners throw open their stores of information to the profession and the public, demand our warmest acknowledgments. From this high source, we have just been favored, through the medium of Dr. Johnson's Journal, with some valuable extracts from an official report by Dr. M'Kinnal, surgeon of the Sybille frigate, "on a Malignant Yellow Fever," which twice visited the crew of that ship

* The whole of Dr. M'Kinnal's Report has not been published. An analysis of it was given in the last Number of the Med.-Chir. Review.—Editor.

with dreadful mortality, on the coast of Africa, in the years 1829 and 1830.

*First Visitation.*

The Sybille had been perfectly healthy from December 1828 to June 1829, cruizing along the coast of western Africa. On the 21st of June, she arrived at Fernando Po, where one or two of her officers only had communication with the shore. Here she received a sergeant and seven marines from the ship Eden, on board of which ship (lately from Sierra Leone,) the yellow fever was still raging, after having carried off her captain, both her surgeons, and fifty of her men. On the 23d, one of the marines received from the Eden fell sick of fever, and was sent on shore. On the same evening the Sybille sailed; and, on the fourth day after having put to sea, a boy on board was seized with yellow fever. He had had no communication with the shore, the Eden, nor, as it is stated, with the sick marine. Six days after this, another case occurred in a marine, who had lately been in the Champion, where yellow fever had also prevailed. Immediately after this attack, the disease broke out in various parts of the Sybille, assumed the most malignant character, and destroyed twenty-two out of sixty-nine cases, which appeared up to the 27th August; when it suddenly ceased. The ship was then to windward of St. Thomas', a most unhealthy island under the line, on her passage to St. Helena, where she arrived, all well, on the 12th September. In all these cases, with two exceptions, the disease was of the continued kind, terminating, in the worst cases, in exhaustion and death, from the third to the sixth day. All classes and colours suffered; the marines most, the negroes least.

*Second Visitation.*

The Sybille sailed again for the coast of Africa, on the 25th October, and continued healthy during the remainder of 1829. On the 3d of January, 1830, she met, at Prince's Island, her own tender, the Black Joke, last from Sierra Leone, where she had been very sickly, and lost twenty-three of her men. All the survivors, however, were "either convalescent or entirely recovered" at the time. Here the Sybille took on board a boy, said to be labouring under "common tertian," which was readily cured. Here also Dr. M'Kechnie, lately from England, assistant surgeon of the Sybille, went, for a few moments, on board the Black Joke, returned to his own ship, and went with her to sea on the 7th of January. Six days after, whilst cruizing off Cape Fermosa, Dr.

M'Kechnie was seized with yellow fever, which soon spread in the ship. He thus became the very first case of the second visitation. The duration of this little epidemic cannot be ascertained from the text, but the malady appears to have attained its acmé early in February, when it became very alarming, and produced the most dreadful havoc. Twenty-six died out of eighty-seven cases.

The interval between this and what is described as a third visitation, appears to have been so short, that the disease can hardly be said to have ceased; as it is stated to have broken out again in St. Helena Roads, on the 22d March, when twenty-two cases occurred, of which six proved fatal. Here also the date of the return to general health is wanting.

Such is the substance of Dr. M'Kinnal's recorded testimony as to the facts of two perfect little epidemics; every case of which ran its course under his own eye, and within the limits of a single ship, of which he was the chief medical officer. This statement is valuable as far as it goes, but it leaves out much on which it would be highly desirable to be more fully informed. For instance, what was the state of public health at Fernando Po, on shore, when the Eden and Champion arrived there, and afterwards when the Sybille anchored there, on the 21st of June? Were the officers, who went on shore from the latter ship, amongst the first attacked in her on going to sea? What were the sanitary measures adopted during the first and second visitations? Were any fresh hands received on board the Sybille, after either of the visitations: and if so, where, when, from whence, how many, of what class, and if recently from England? Were these fresh hands the first attacked on each occasion, and had they ever had the disease before? In what did the difference consist between the last cleaning given to the Sybille by the negroes, and the former cleanings which she must have undergone? How long did the ship remain in yellow-fever latitudes, after her last visitation? Where did she touch, and what communications had she with infected persons, places, or things? Is there any well-authenticated case of an individual who had passed the disease, in the first visitation, having been attacked in the second or third? The want of information on these, and many other important particulars, leaves a deplorable break in the chain of circumstantial evidence.

When a medical man brings before the public the history of an epidemic in which he has been personally engaged, with a view to elucidate the questions of contagion and monopathy of a particular disease, he stands before his professional brethren, as a witness does in presence of a jury, in a court of justice. As long as either details plain facts, attested by his own senses, he is listened to with attention, because he is furnishing to the jury the very materials on which their verdict is to be founded: but when either ceases to be the relator of what he has seen, and offers his own inferences and speculations, instead of actual occurrences, he lays down the sacred office of a witness, and takes up that of an advocate. A shade of distrust is forthwith interposed, and the jury stands aloof from the evidence which would seem inclined to dictate to their judgments.

Dr. M'Kinnal, to whose zeal and candour I pay a willing tribute, because I know it to be deserved, must not be offended when I say, that I consider his inferences as to the origin and contagious nature of the yellow fever on board the Sybille, to be in direct opposition to his own testimony. His conclusions are but two in number, at least there are but two that bear directly on the history of the Sybille epidemics; viz.

1st. That the disease originated, in all the visitations, on board the ship itself, from noxious vapours, and from decomposition of the wood under the limber-boards and lining of the vessel.

2. That the disease was not contagious.

If there ever were proof of the importation of disease from one ship into another, of its communication from one set of persons to another, by personal infection, or contagion if you will, we have that proof in Dr. M'Kinnal's facts. A ship is perfectly healthy for six months, in a certain climate, and, without quitting that climate, is attacked with yellow fever, four days after she had received eight men from another ship, in which the disease was actually raging at the time the transfer was made. This was in June, at Fernando Po and Prince's Island, a little to the north of the equator. During July and August, the ship sailed southward, and crossed the line. No doubt every exertion that skill, discipline, and zeal could use, was vigorously applied to arrest the spread of the malady. It ceased about the beginning of September. But, as if to prove that neither season nor situation could alone produce it, the ship returned from St. Helena in October, cruised again on the coast of Africa, on her old ground, and continued healthy for three months, when, in January, her assistant surgeon, fresh from England, went on board an infected ship, returned to his own, in which he put to sea, was attacked with yellow fever on the sixth day after his imprudent intercourse, and again the Sybille became the scene of disease and death. Again the ship crossed the equator for

St. Helena, and again the disease ceased, "after a thorough cleaning out, succeeded by ventilation and all kinds of purification."

The grounds on which Dr. M'Kinnal, apparently concludes that the yellow fever on board the Sybille was not contagious, are,

1. That many escaped without any attack of the disease.
2. That, after having reached its acmé, it did not continue to spread.
3. That it could not be traced from man to man, nor from mess to mess.

A little reflection will show that the two first of these arguments bear as strongly against local infection, as they do against personal contagion, and are therefore of no value on either side.

As to the third argument, we have instances of the spread even of plague, and even smallpox, having been arrested by careful sanitary regulations on board ships in which many unprotected persons remained still unattacked, whilst those who were attacked did not stand at all in the order of proximity to the sick, the centre of contagion.

The following extract from the journal of Mr. Little, surgeon of his Majesty's ship Atlas, dated off Cadiz, Sept. 1809, is too valuable to be omitted.

"One complaint, which at first became a little alarming, was the smallpox, as a great number of the ship's company were found not to have had that disease. It was at first brought on board by the boat's crew from Cadiz; yet fourteen cases only took place, and all of them recovered on board. But with the unremitting and vigorous exertions that were used in detaching them from the ship's company, making the people bathe every morning, and keeping the ship dry, and as well ventilated as possible, an effectual stop was put to its progress on board; a circumstance scarcely to be expected with such a contagious disease: yet it demonstrates, under these kinds of impending dangers, what may be done by judicious and well-regulated economy, in the internal arrangement of a ship."

Dr. Burnett's very remarkable official report to the Lords of the Admiralty, as to the infection with yellow fever, or African fever, of the garrison of the Island of Ascension, in 1823, by the Bann sloop of war, and as to the subsequent infection of the Driver sloop at the same place; the Tartar frigate at Bahia* by the same ship, ought to remove all

* Vide Official Report, p. 16.

doubts, if any serious doubts still exist, as to the possibility of this disease being transplanted from place to place, and from ship to ship, after the manner of the most contagious diseases, with some limitation as to climate and season, but with none as to distance.

The alarming fact, now but too evident, that the seeds of exotic disease may be imported and propagated amongst us like the seeds of exotic plants, and the ova, or parent stock of animals, must at this moment be peculiarly interesting to Europe, where every man enjoys almost an ubiquity of residence by means of steam, and where we are threatened on all sides by the rapid march of pestilence.

Let me conclude, sir, by expressing a hope that Dr. M'Kinnal will, for the sake of humanity and science, take an early opportunity of noticing the queries contained in this letter, and of rendering his report more satisfactory to the profession, by every additional elucidation in his power to afford.

I remain, sir, your very obedient servant,

SANITARIUS.

*London; 24th November,* 1830.

---

