# Errata in the Last Number

**Published:** 1831-02

**Authors:** 


**Errata** ***in the last Number.***

**P. 21, line 24, for "if there even were proof," read "if there *ever* were proof."**
**22, — 2 from the bottom, for "of the same place," read "*at* the same place."**

